# Gold Nanorod Assemblies: The Roles of Hot-Spot Positioning and Anisotropy in Plasmon Coupling and SERS

**DOI:** 10.3390/nano10050942

**Published:** 2020-05-14

**Authors:** Priyanka Dey, Verena Baumann, Jessica Rodríguez-Fernández

**Affiliations:** 1 Department of Physics and CeNS, Ludwig-Maximilians-Universität München, 80539 Munich, Germany; verena-baumann@gmx.de (V.B.); jessica.rodriguezfdez@gmail.com (J.R.-F.); 2 Nanosystems Initiative Munich (NIM), 80799 Munich, Germany

**Keywords:** gold nanorods, silver coating, DNA linker, core–satellite nanoassembly, directional assembly, anisotropy, hot-spot positioning, SERS molecule positioning

## Abstract

Plasmon-coupled colloidal nanoassemblies with carefully sculpted “hot-spots” and intense surface-enhanced Raman scattering (SERS) are in high demand as photostable and sensitive plasmonic nano-, bio-, and chemosensors. When maximizing SERS signals, it is particularly challenging to control the hot-spot density, precisely position the hot-spots to intensify the plasmon coupling, and introduce the SERS molecule in those intense hot-spots. Here, we investigated the importance of these factors in nanoassemblies made of a gold nanorod (AuNR) core and spherical nanoparticle (AuNP) satellites with ssDNA oligomer linkers. Hot-spot positioning at the NR tips was made possible by selectively burying the ssDNA in the lateral facets via controlled Ag overgrowth while retaining their hybridization and assembly potential at the tips. This strategy, with slight alterations, allowed us to form nanoassemblies that only contained satellites at the NR tips, i.e., directional anisotropic nanoassemblies; or satellites randomly positioned around the NR, i.e., nondirectional nanoassemblies. Directional nanoassemblies featured strong plasmon coupling as compared to nondirectional ones, as a result of strategically placing the hot-spots at the most intense electric field position of the AuNR, i.e., retaining the inherent plasmon anisotropy. Furthermore, as the dsDNA was located in these anisotropic hot-spots, this allowed for the tag-free detection down to ~10 dsDNA and a dramatic SERS enhancement of ~1.6 × 10^8^ for the SERS tag SYBR gold, which specifically intercalates into the dsDNA. This dramatic SERS performance was made possible by manipulating the anisotropy of the nanoassemblies, which allowed us to emphasize the critical role of hot-spot positioning and SERS molecule positioning in nanoassemblies.

## 1. Introduction

Plasmonic nanostructures featuring control over their morphology and tunable optical properties, especially tunable localized surface plasmon resonance (LSPR) and surface-enhanced Raman scattering (SERS), are of interest to nanomaterial scientists [1,2,3,4]. The coupled LSPR mode appears when plasmonic nanoparticles (NPs) are close to each other, resulting in coupling of the individual plasmon oscillations via near-field interactions [5]. This coupling strongly impacts the electric field distribution around the nanostructure, especially at the NP–NP junctions, or “hot-spots”. When molecules are in close proximity to regions of such high electric field hot-spots, their Raman signal intensities are dramatically enhanced, which is referred to as SERS [6]. This has motivated the development of plasmonic nanoassemblies and their use as SERS agents. Colloidal nanoassemblies with nanogaps of 0–10 nm are capable of strong SERS enhancements in the order of 10^2^–10^10^ and have inspired researchers to investigate novel ways to fabricate them [2,7,8]. Such nanoassemblies have found extensive applications as SERS nano-, bio-, and chemosensors [9,10] for various analytes, and more recently as biomedical diagnostic agents [11,12]. An important criterion for biomedical SERS agents which allows diagnosis by tracking the SERS tag signal is utilization of the near-infrared (NIR, 650–900 nm) region. To this end, NIR lasers have gained popularity due to their higher light penetration depth and low cellular damage induced. This has strongly fostered the development of NIR-active plasmonic nanostructures [4,13,14].

Sub-100 nm NIR-active plasmonic colloidal nanoassemblies have a high potential for use in biomedical diagnosis, thanks to the strong sensitivity boost that precisely controlled plasmonic coupling effects can provide [15,16]. However, the preparation of plasmon-coupled nanoassemblies with strict control over the assembly morphology and hot-spot NP–NP junction formation still remains quite challenging. In this regard, fabrication of various plasmonic nanoassemblies has been attempted. Among them, core–satellite nanoassemblies [5,17] have a unique arrangement, providing significantly better hot-spot control than other assemblies, such as globular assemblies, nanochains, and nanobranches [18,19]. Linker molecules that mediate the formation of such nanoassemblies and control the hot-spots (density and position) are, thus, significant. Tailored polymer linkers, such as dendrimers or hyperbranched polymers [18], do not provide the site-selectivity needed for the precise positioning of the NPs that translates into precise positioning of the hot-spots. This issue can be overcome with other linker types, where DNA particularly stands out. Its high programmability enables the preparation of complex-shaped all-DNA nanostructures, employing the DNA origami technique [20]. Such nanostructures can serve as templates for the self-assembly of plasmonic NPs, allowing for a tight control over the NP placement position, and hence over hot-spot positioning. However, the NP–NP distances featured by these types of nanoassemblies are typically large (>6–8 nm) [21,22], which leads to weak plasmonic coupling and poor SERS performance. In this regard, template-free strategies relying on the simple utilization of short ssDNA oligomers as linker molecules can be advantageous. Complementary ssDNA hybridization can facilitate NP hot-spot positioning in ssDNA-mediated nanoassemblies. However, controlling the hot-spot density and precisely positioning the hot-spot, as well as positioning the SERS-active molecule in that intense hot-spot, are significant hurdles. When controlled, these factors would maximize the SERS signal. The use of gold nanorod assemblies with ssDNA as both the linker and SERS molecule would, thus, be an ideal nanostructure to investigate the importance of the hot-spot positioning and anisotropy, which have not yet been studied systematically.

For the formation of strongly coupled nanoassemblies with ssDNA-functionalized AuNRs, a key challenge is driving site-selective hot-spot formation at the nanorod tips, i.e., providing anisotropy or directionality where the electric field enhancement is inherently maximal. This is not straightforward, as any linker such as ssDNA functionalizes the AuNR identically at both the lateral facets and the tips, making the AuNR@DNA isotropic in behavior, i.e., exhibiting the identical hybridization potential of the ssDNA located at both the NRs’ tips and lateral facets [23]. Very selective attempts have been made to this end to preserve the anisotropic behavior of gold nanorods in gold nanorod assemblies with AuNR core and AuNP satellites. Xu and co-workers [24] employed differential surfactant–gold surface affinity to achieve this. They selectively grafted two distinctive ssDNA sequences at their tips and sides through a cetrimonium bromide CTAB-assisted blocking strategy, and suggested that a complex trade-off between surfactant concentration and the AuNRs-to-ssDNA ratios, along with degrees of ssDNA surface functionalization, helped in maintaining the anisotropy in the nanoassemblies. They further reported that a higher coverage of ssDNA on the AuNR led to higher satellite coverage, and therefore higher hot-spot density, which resulted in higher SERS performance. Higher hot-spot density, in their case, was obtained for non-directional nano-assemblies rather than directional anisotropic nano-assemblies. Thus, their efforts toward retaining the anisotropic nature (end-to-end assemblies) in the nanoassemblies was not rewarding for SERS performance. Their observation could possibly be due to the use of an external SERS molecule 4-aminothiophenol (4-ATP), which does not specifically sit in the intense hot-spot region, whereas the net 4-ATP concentration and available nanosurface area contribute to the resultant SERS performance. The tag was incorporated after formation of the nanoassemblies, which is a rather common method. However, addressing the SERS incorporation protocol, Dey et al. [25] have reported that incorporation of the SERS tag before creation of the NP–NP junction hot-spot allowed for better SERS performance than the typical method, where tag incorporation occurs post-nanoassembly formation. They employed 2-quinolinethiol (QTH) tags with spherical core–satellite nanoassemblies for their study. Therefore, an understanding needs to be developed of the hot-spot density and positioning, and the plasmon coupling with that of the SERS molecule positioning and SERS performance. This knowledge could guide the community to tailor their nanoassembly formation methodology to potentially push the SERS enhancement limits another step further.

We here report a strategy to avoid multiple satellites randomly anchoring onto the core AuNR, instead limiting the satellites to the NR tips where the electric field, i.e., hot-spot intensity, is inherently highest. This was achieved by selectively burying the linker ssDNA at the lateral facets via Ag overgrowth on the AuNR, such that the ssDNA retain the activity or hybridization potential only at the NR tips. Most importantly, we demonstrate the crucial role that the extent of burial of embedded or inactivated DNA has on yielding anisotropic nanoassemblies, thereby greatly affecting the plasmon coupling and SERS performance. We developed a strategy to maintain the anisotropy or directionality of the gold nanorod structure in customized gold nanorod assemblies. This methodology, therefore, serves as a robust and scalable approach to readily obtain plasmon-coupled nanoassemblies with precisely positioned hot-spots and optically critical anisotropic nature. We further illuminate the crucial role of the directional hot-spots, i.e., SERS molecules (tags) positioned in hot-spots with both intrinsic (inherent) and extrinsic (external) SERS tags, in an attempt to extract higher plasmon coupling and SERS from such plasmonic nanoassemblies.

## 2. Materials and Methods

***Chemicals.*** Hydrogen tetrachloroaurate trihydrate (HAuCl_4_·3H_2_O), sodium borohydride (NaBH_4_), silver nitrate (AgNO_3_), and cetyltrimethylammonium bromide (CTAB, >98%) were purchased from Sigma-Aldrich (St. Louis, MO, USA). Magnesium chloride (MgCl_2_, >99%) was procured from Roth, polyethylene glycol (PEG) α-methoxy-ω-mercapto (HS-PEG-OMe, MW 5000 Da) from Rapp Polymere, and sodium oleate from TCI America (Portland, OR, SUA). Tetrahydrofuran (THF, >99.9%) was ordered from Merck (Darmstadt, Germany). The 6-carboxyfluorescein (6-FAM) modified (5′-thiol-C6-TTTTTTTTTTTTTTTTTT-3′-6-FAM) (hereafter T_18_-DNA; 200 μM in water), (5′-thiol-C6-AAAAAAAAAAAAAAAAAA-3′-6-FAM) (hereafter A_18_-DNA; 200 μM in water), and 5′-thiol-C6-PEG-TTTTTTTTTTTTTTTTTT-3′-6-FAM (hereafter, PEG-T_18_-DNA) were procured from Biomers.net (Ulm, Germany). Tris–borate–EDTA buffer (1× TBE, pH 8.0) and Tris–acetate–EDTA buffer (1× TAE, pH 8.0) were also used. Silver nitrate and hydroquinone was obtained from Sigma (St. Louis, MO, USA) and used as received. SYBR gold was obtained from ThermoFischer (Dreieich, Germany). All chemicals were used without further purification. In all preparations, ultrapure Milli-Q water (Merck, Darmstadt, Germany) (H_2_O, 18.2 MΩ cm) was used.

***DNA functionalization of AuNRs (AuNRs@DNA).*** CTAB-capped AuNRs measuring 74 ± 7 nm in length and 21 ± 3 nm in width with an aspect ratio ~3.5 were synthesized as reported earlier [26,27]. The single-crystal NRs were functionalized with single-stranded DNA, as described recently by us [28]. For this, the AuNRs@CTAB were functionalized with poly(ethylene glycol) (MeO-PEG-SH, added dose ~20 PEG chains/nm^2^) and ultimately redispersed in THF, as described therein. Functionalization with 6-mercaptohexanoic acid (MHA) allowed for back-transfer into TBE 1× buffer and subsequent functionalization with ssDNA, namely HS-T_18_-6-FAM (T_18_-DNA hereafter, as in the main text), at a dose of ~1 ssDNA strand/nm^2^, which yielded AuNRs@DNA with a grafting density of ~727 DNA strands/NR. As demonstrated in our earlier work [28], the AuNRs@DNA prepared this way are very stable in buffer and biological media. They are also highly biocompatible due to their robust colloidal stability and efficient CTAB removal during the functionalization process. The AuNRs@DNA were redispersed in TAE 1× containing 0.001% Tween 80. The transverse and longitudinal plasmon bands of the AuNRs@DNA were centered at ~510 and ~790 nm, respectively.

***Formation of anisotropic directional AuNRs@DNA@Ag building blocks by selective DNA inactivation of isotropic AuNRs@DNA.*** DNA inactivation was carried out through the controlled overgrowth of Ag on the single-crystalline AuNRs@DNA by reduction of AgNO_3_ assisted by hydroquinone. The optimal overgrowth conditions were obtained by carefully adjusting several experimental parameters and is detailed in Supplementary Materials Section S1. In the typical Ag overgrowth experiment, AuNRs@DNA was first redispersed in TAE 1× containing 2% Tween 80. To this dispersion, a few µL of a 2 M NaCl stock solution was added, followed by vortexing in order to yield the following final concentrations: Au = 0.15 mM and NaCl = 0.05 M. Thereafter, 4.3 μL of an aqueous 0.4 mM AgNO_3_ solution was added. This was followed by the quick injection of 4.3 μL of an aqueous 0.4 mM hydroquinone solution under sonication and subsequent vortexing. The silver overgrowth process was monitored by UV–Vis–NIR spectroscopy. The Ag^+^ reduction was typically completed within 50–70 min, depending on the DNA grafting density on the AuNRs@DNA. Directional building blocks with lateral side-blocked (Ag-buried) DNA strands were obtained at optimal reaction times (typically ≥40 min), depending on the length of the DNA strands in the AuNRs@DNA. In order to quench the reaction, 2500 µL of TAE 1× buffer containing 4% Tween 80 was quickly injected into a 500 µL AuNRs@DNA@Ag colloid. Subsequently, this was left undisturbed for approximately 30 min in order to allow physisorption of the Tween 80 onto the Ag shell surface and thereby act as a stabilizer. It was noted that this physisorption step was very important to prevent the aggregation of the AuNRs@DNA@Ag during the subsequent centrifugation or redispersion cycles. Washing was carried out upon centrifugation at 5000 rpm for 10 min, followed by resuspension in TAE 1× containing 2% Tween 80.

***Formation of anisotropic directional nanoassemblies with anisotropic AuNRs@DNA@Ag as building blocks.*** For the formation of directional nanoassemblies, we utilized the ssDNA hybridization affinity of thymine T_18_-DNA with adenine A_18_-DNA. In a typical experiment, 600 μL of directional AuNRs@DNA@Ag building blocks in TAE 1× containing 2% Tween 80 (and having T_18_-DNA strands in this case) was mixed in an Eppendorf tube with 108 μL of 15 nm spherical AuNPs@DNA (stock [Au] = 0.8 mM) functionalized with complementary A_18_-DNA strands. NaCl 2M was then added so as to reach a net NaCl concentration of 0.07 M. We note here that a lower NaCl concentration was found to be insufficient to screen the strong electrostatic repulsion between the T_18_-DNA and the A_18_-DNA strands, thus hindering their hybridization and in turn leading to a low assembly yield. On the other hand, NaCl concentrations higher than 0.7 M resulted in significant aggregation of the AuNRs@DNA@Ag. Assembly was allowed to proceed overnight under gentle shaking in an orbital shaker. Nonhybridized AuNPs@A_18_-DNA were removed by centrifugation at 10,000 rpm for 10 min and resuspended in TAE 1× containing 2% Tween 80.

***Formation of nondirectional nanoassemblies with nondirectional isotropic AuNRs@DNA@Ag as building blocks.*** The formation of nondirectional nanoassemblies was performed as described above for directional nanoassemblies. The only difference is that nondirectional nanoassembly formation was triggered by utilizing building blocks of nondirectional isotropic AuNRs@DNA@Ag (for which the Ag thickness on the lateral facets is lower than the actual DNA length). In our case, we utilized two types of nondirectional building blocks: (i) AuNR@DNA@Ag (with the DNA strands being T_18_-DNA) obtained at a Ag overgrowth reaction time of 30 min; and (ii) AuNR@PEG-DNA@Ag (with the PEG-DNA strands being PEG-T_18_-DNA) overgrown for 50 min, to further form nanoassemblies.

***Surface-enhanced Raman spectroscopy (SERS) tagging of directional nanoassemblies.*** In order to further scrutinize the importance of SERS molecule positioning at the very intense and precisely positioned hot-spot, we utilized an external SERS tag SYBR gold, which has high affinity for binding to dsDNA. As the dsDNA is only formed at the tips or precisely positioned at the hot-spots for anisotropic directional assemblies, the SERS tag can only sit at those intense and precisely-positioned hot-spots. This allowed us to contemplate the relation between the hot-spot density and positioning, and the plasmon coupling with that of SERS molecule positioning and SERS performance, which had not been investigated previously. Thus, an external SERS tag (namely SYBR gold, from Thermo Fischer, Dreieich, Germany) was employed. For tagging, the commercially-supplied 10,000× (≡19.6 mM) [29] SYBR gold stock was first diluted in TAE 1× buffer containing 0.0001% Tween to yield a SYBR gold stock with a 10× concentration (≡19.6 µM). Then, 30 μL of this SYBR gold stock was added under vortexing to 20 μL of the directional nanoassemblies redispersed in TAE 1× containing 0.0001% Tween 80. An additional dilution step was performed by addition of 50 μL TAE (1×) containing 0.0001% Tween. This resulted in a net concentration of 3× (≡0.58 µM) of SYBR gold in the tagged directional nanoassemblies. The intercalation of the SYBR gold dye molecules into the dsDNA was allowed to proceed overnight. This also allowed us to determine their enhancement factor (see calculations in Section S2.4 in Supplementary Materials).

***Transmission electron microscopy (TEM).*** TEM was carried out in a JEOL 1010 (Tokyo, Japan) operating at 100 kV. Grid preparation for TEM examination of AuNRs@DNA, AuNRs@DNA@Ag, and of the different nanoassemblies was carried out. Upon drop-casting a concentrated colloidal dispersion and subsequent solvent evaporation, complete oxidation (disappearance) of the Ag layer on the AuNRs@DNA@Ag was observed. In this regard, we found that the best way to minimize this effect was to allow for 5 min contact between an oxygen plasma-activated TEM grid and the colloidal dispersion under investigation, followed by complete removal of the excess colloid with a filter paper. This resulted in a low density of either nanoparticles or nanoassemblies on the TEM grid. Therefore, a carbon-coated Cu TEM grid was activated with O_2_ plasma for 30 s. Thereafter, 5 μL of the corresponding colloidal sample was drop-casted onto it. After 5 min contact, the excess colloid was removed using a Whatman filter paper. Finally, the grid was carefully dried with a N_2_ gun.

***UV–Vis–NIR spectroscopy.*** All UV–Vis–NIR extinction spectra were measured in a Cary 5000 UV–Vis–NIR spectrophotometer using 2 mm pathlength quartz cuvettes.

***Surface-enhanced Raman spectroscopy (SERS).*** The SERS performance of directional AuNRs@DNA@Ag building blocks and their corresponding directional nanoassemblies was investigated in a dark-field microscope (DFM, Zeiss) coupled to a CCD Raman spectrometer (Princeton Instruments Acton SP2500 Spectrometer, liquid nitrogen-cooled, Trenton, NJ, SUA) with a 633 nm laser line. A 633 nm laser line, being close to the NIR optical window, would be beneficial for use in biomedical applications, and hence was our choice. The samples were prepared for SERS analysis as follows. First, glass cover slips measuring 24 × 24 mm^2^ were cleaned in an ultrasonic bath for 10 min in acetone, followed by 10 min in isopropanol, 10 min in 2% Hellmanex, and 10 min in Milli-Q water. The cleaned glass slides were then dried with a N_2_ flow. The corresponding colloids (either the anisotropic directional AuNRs@DNA@Ag, their anisotropic nanoassemblies, and the SYBR-gold-tagged anisotropic directional nanoassemblies) were conveniently diluted in order to obtain a low density per µm^2^ (∼0.15, as determined from SEM) on the glass substrate upon spin-coating, ensuring that only individual, well-separated AuNRs@DNA@Ag and directional nanoassemblies were investigated by SERS. For this, 50 μL of a diluted dispersion of the corresponding colloid were spin-coated at 1000 rpm for 30 s onto the clean substrates. The substrates were carefully dried with a N_2_ gun. For SERS analysis, we first identified individual scattering spots under dark-field illumination. Thereafter, a 633 nm laser was focused onto the given spot and the corresponding Raman spectrum was acquired for 5 min. In addition to the main text, additional SERS spectra, SERS detection limits, and enhancement calculations are provided in Supplementary Materials Section S2.

## 3. Results and Discussion

**Figure 1:**
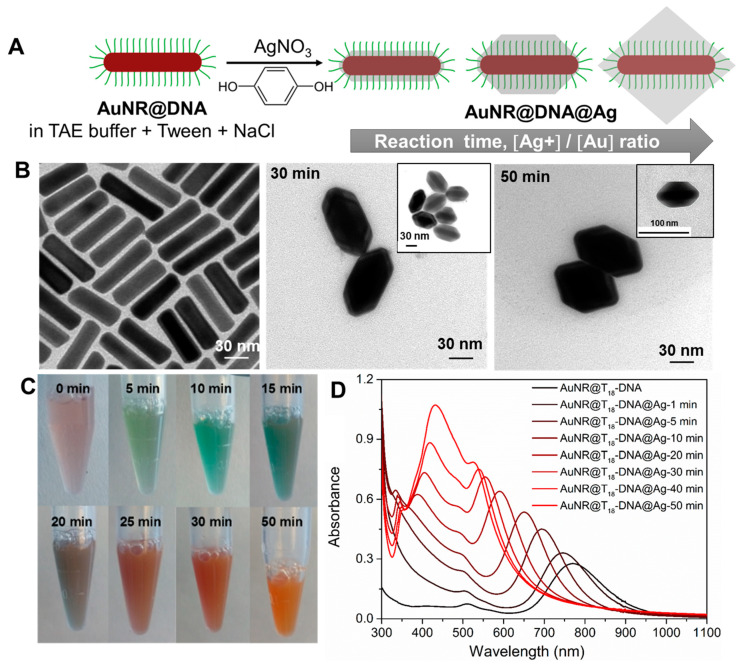
(**A**) Sketch showing the gradual evolution of AuNRs@DNA into directional building blocks by epitaxial Ag overgrowth. By controlling the reaction time and Ag^+^/Au ratio, the resulting AuNRs@DNA@Ag will be: nondirectional (truncated octahedra with all DNA strands available for hybridization, left); directional (truncated octahedra with the DNA strands at the tip region available for hybridization, center); or partially directional, but colloidally unstable (nontruncated octahedra with most DNA strands inactivated for hybridization, right). (**B**) TEM micrographs depicting the starting AuNRs@T_18_-DNA (0 min, left panel) and the resulting AuNRs@T_18_-DNA@Ag at an intermediate reaction time (30 min) and after completion of the reaction (50 min). (**C**) Photographs illustrating the color changes of the AuNRs@T_18_-DNA dispersion (0 min) at the indicated reaction times. (**D**) Corresponding UV–Vis–NIR spectra during Ag overgrowth.

***Formation of Anisotropic directional building blocks of AuNRs@DNA@Ag using selective DNA inactivation of isotropic AuNRs@DNA.*** Here, we present a robust strategy to render AuNRs@DNA with directional interactions at their tip region by simply and selectively inactivating the ssDNA grafted on their lateral facets for DNA hybridization. This was achieved by strategically burying the DNA of the lateral facets into a Ag overgrown shell. We carefully designed our AuNR@DNA@Ag building block so that we could utilize two important behaviors: (i) Ag overgrows epitaxially on single-crystal AuNRs [30] or Au truncated octahedrons [31]; and (ii) Ag can be overgrown on AuNPs regardless of ssDNA being grafted on their surface [32]. We show that single-crystal AuNRs@DNA can undergo an identical Ag overgrowth process under optimized experimental conditions, leading to the formation of AuNRs@DNA cores encapsulated by an octahedrally-shaped Ag shell, as schematically shown in Figure 1A. As will be shown, a precise control over the truncation extent of the AuNRs@DNA@Ag octahedra is a powerful tool to provide the non-directional isotropic AuNRs@DNA with highly directional interactions, therefore serving as a robust and scalable approach to readily obtain plasmonically-coupled nanoassemblies with precisely-positioned hot spots.

As starting building blocks, we synthesized single-crystal CTAB-capped AuNRs measuring 74 ± 7 nm in length and 21 ± 3 nm in width, and with an average aspect ratio of ~3.5 [26,27]. The AuNRs were functionalized with ssDNA, namely with a 5′-thiol-C6-T_18_-3′-6-FAM sequence (T_18_-DNA), as recently reported by us [28], leading to the dispersion of highly stable, biocompatible, CTAB-free AuNRs@DNA in Tris-acetate-EDTA (TAE) 1× buffer containing 0.001% Tween 80. We pursued the selective inactivation of the ssDNA grafted on the lateral facets of AuNRs@DNA by overgrowing Ag on their surfaces. Our first overgrowth approach was inspired by the work of Lee et al. [33], who showed that Ag^+^ can be reduced to Ag^0^ on the surfaces of DNA-functionalized spherical AuNPs, assisted by polyvinylpyrrolidone (PVP) and L-sodium ascorbate. The translation of this approach to AuNRs@DNA yielded high quality AuNRs@DNA@Ag. However, the PVP was shown to negatively interfere with DNA hybridization during subsequent assembly (see Figures S1 and S2, and Section S1.1 in Supplementary Materials); hence, this approach was discontinued. In contrast, we observed that the reduction of Ag^+^ assisted by hydroquinone (HQ), a method earlier deployed by Sánchez-Iglesias et al. for the epitaxial deposition of Ag onto water-dispersible single-crystal SH-PEG-capped AuNRs [30], yielded homogeneous AuNRs@DNA@Ag under optimal buffer, ionic strength, and reactant ratio conditions (see Section S1.2 in Supplementary Materials). In a typical experiment, AuNRs@T_18_-DNA with a DNA grafting density of ~727 DNA strands/NR was redispersed in a TAE buffer solution (pH = 8.0) containing 2% Tween 80 and 0.05 M NaCl, so that Au = 0.15 mM. Ag overgrowth was initiated with the addition of AgNO_3_ in a Ag^+^/Au ratio of 6, followed by the quick addition of an aqueous HQ solution in a ratio HQ/Ag^+^ = 1. The morphological evolution of the AuNRs@T_18_-DNA was followed by TEM (> 100 particles analyzed with ImageJ) and monitored in situ by UV–Vis–NIR spectroscopy at different reaction times until no further optical changes were recorded. Figure 1B illustrates the gradual evolution of the initial AuNRs@T_18_-DNA (Figure 1B, left panel) into well-defined truncated AuNRs@T_18_-DNA@Ag octahedra, featuring an average silver thickness of ~3–5 nm at their tips, and average thicknesses at their lateral facets of ~14 ± 2 nm after 30 min reaction (Figure 1B, center) and of ~20 ± 2 nm upon completion of the reaction after 50 min under the above experimental conditions (Figure 1B, right panel). The preferential deposition of Ag in the lateral facets of AuNRs@DNA is likely driven by the blocking role that T_18_-DNA (which bears an end thiol functionality) at the AuNRs tips (i.e., inherent property of {100} crystal facet). In this sense, the mercapto-functional DNA sequence seems to work in a similar fashion as SH-PEG, as reported by Sánchez-Iglesias et al. [30], effectively blocking Ag growth along the AuNRs’ longitudinal direction, and therefore leading to their gradual evolution into AuNRs@DNA@Ag octahedra.

The quality and stability of the AuNRs@DNA@Ag is critically affected by several factors (see Figures S3–S7 and associated discussion in Section S1.2, SI). For instance, unlike for AuNRs@PEG, we found that for AuNRs@DNA both NaCl and Tween 80 are necessary for the controlled epitaxial overgrowth of Ag. NaCl plays a critical role in the quality of the epitaxial overgrowth, while Tween assists in preventing the aggregation of the growing AuNRs@T_18_-DNA@Ag (Figures S6 and S7). Additionally, performing the overgrowth in buffers, such as PBS, led to the precipitation of inorganic silver phosphate species and changes in the reaction kinetics, resulting in an uncontrolled and inhomogeneous Ag overgrowth (Figures S3–S5). All these issues were successfully overcome by using TAE as buffer. We also note that at Ag^+^/Au ratios >6, nontruncated AuNRs@T_18_-DNA@Ag octahedra are obtained upon reaction completion. Under those conditions, the T_18_-DNA strands are almost completely buried in Ag (see Figure 1A, right-most cartoon). Since the nonburied strands are not able to stabilize them in the buffer medium, aggregation occurs in the long term. For this reason, in this work we have focused on the colloidally stable truncated AuNRs@T_18_-DNA@Ag octahedra obtained for Ag^+^/Au = 6 at intermediate reaction times (Figure 1A—left cartoon) and upon reaction completion (Figure 1A—center). During Ag overgrowth, the colloidal dispersion of AuNRs@T_18_-DNA gradually changes color from pale brown into orange (see photographs in Figure 1C), reflecting the spectral evolution during the process (Figure 1D). The longitudinal plasmon band of the starting AuNRs@T_18_-DNA at ~790 nm (black curve in Figure 1D) undergoes a gradual blue shift upon silver deposition due to the steady decrease in aspect ratio. After approximately 20 min reaction, a plasmon band at ~410 nm appears due to the significant Ag thickness in the lateral facets. It further red-shifts and gains in intensity as the reaction continues and reaches completion after 50 min. It is also noteworthy that the narrow peak at ~334 nm becomes apparent after about 10 min reaction and it gradually red-shifts (up to ~350 nm), broadens, and decreases in intensity as the silver overgrowth proceeds. This mode may correspond to a high-order plasmon resonance, likely of octupolar nature, as reported for similar Au-Ag core–shell nanorods by Jiang et al. [34]. ImageJ was used to analyze 100–115 nanostructures to better understand their dimensions. The average Ag thickness in the lateral facets of the AuNRs@T_18_-DNA@Ag overgrown for 50 min was ~20 ± 2 nm, while the estimated length of the T_18_-DNA sequence assuming a stretched conformation was ~16 nm [35]. Hence, the T_18_-DNA strands in the lateral facets were expected to be fully inactivated for hybridization as a result of being embedding into the Ag coating, thereby morphologically retaining the tip-active functional anisotropic nature of the optically active gold nanorods.

**Figure 2:**
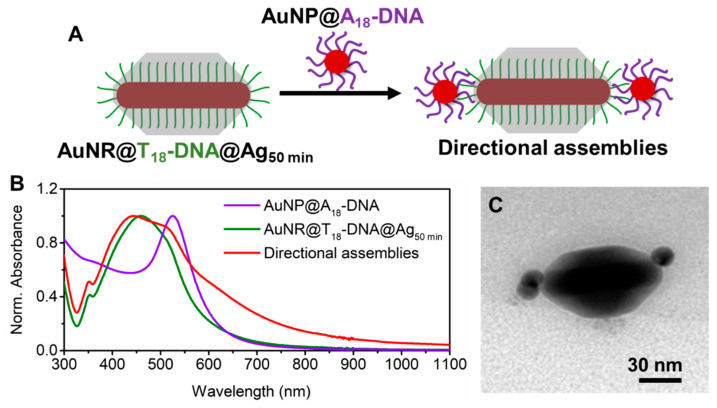
(**A**) Schematic illustration of the formation of anisotropic directional nanoassemblies upon incubation of AuNRs@T_18_-DNA@Ag (overgrown for 50 min) with AuNPs@A_18_-DNA. (**B**) UV–Vis–NIR spectra of each building block and the resulting directional anisotropic nanoassemblies. (**C**) Representative TEM micrograph of the obtained anisotropic directional nanoassemblies.

**Figure 3:**
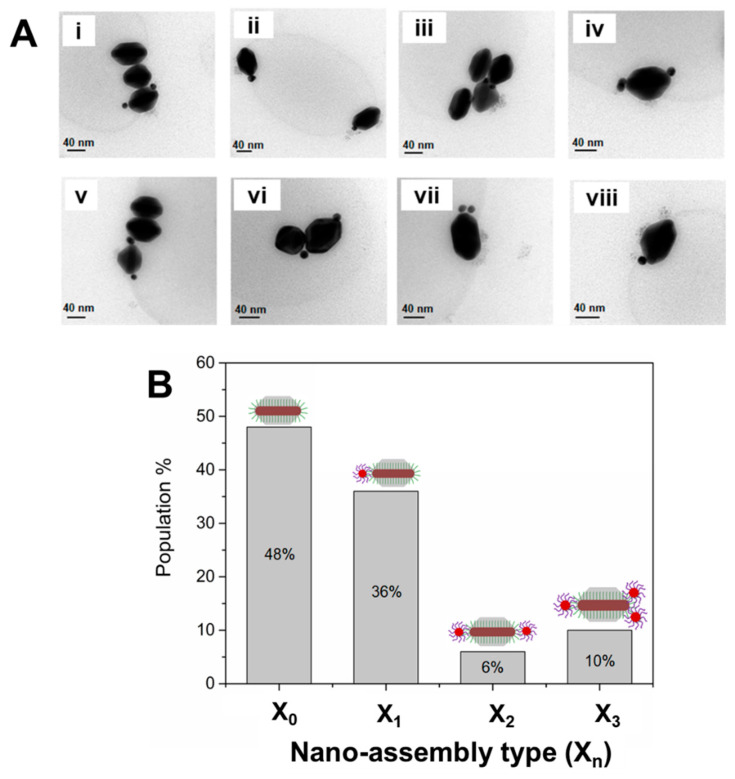
(**A**) (i–viii) Representative TEM micrographs of the anisotropic directional nanoassemblies obtained upon incubation of core AuNRs@T_18_-DNA@Ag (overgrown for 50 min) with satellite AuNPs@A_18_-DNA. (**B**) Nanoassembly yield (determined from statistical TEM analysis, population count = 100), resulting from the DNA hybridization of AuNRs@T_18_-DNA@Ag (overgrown for 50 min) with AuNPs@A_18_-DNA as a function of nanoassembly type X_n_, where *n* = number of spherical satellite NPs per assembly. Overall, the anisotropic directional nanoassembly yield was ~52% (X_1_, X_2_, X_3_).

***Formation of Anisotropic directional nanoassemblies with anisotropic AuNRs@DNA@Ag as building blocks.*** Truncated AuNR@DNA@Ag with optimal dimensions prepared as described above can display site-selective DNA interactions in the tip regions, as long as the DNA strands grafted at the AuNRs’ lateral facets are effectively buried by the deposited Ag. To test this, we incubated the AuNRs@T_18_-DNA@Ag overgrown for 50 min (see Figure 1B) with spherical AuNPs (~15 nm) functionalized with a 5′-thiol-C6-A_18_-3′-6-FAM (A_18_-DNA) sequence, which was complementary to the T_18_-DNA one. Assembly formation was allowed to proceed overnight under gentle shaking and in the presence of 0.07 M NaCl to allow for charge screening. The excess AuNPs@A_18_-DNA was removed by centrifugation (see Materials and Methods). This resulted in directional nanoassemblies upon hybridization with the AuNPs@A_18_-DNA (see sketch in Figure 2A). The formation of directional anisotropic nanoassemblies was confirmed by UV–Vis–NIR spectroscopy and TEM. The extinction spectra in Figure 2B indicate a significant plasmon coupling in the nanoassemblies (red curve) as compared to the spectrum of the AuNRs@T_18_-DNA@Ag (green curve) and AuNPs@A_18_-DNA (violet curve) building blocks. This confirms that successful DNA hybridization occurred between T_18_-DNA strands in the tip region of the AuNRs@T_18_-DNA@Ag and A_18_-DNA region of the AuNPs@A_18_-DNA. The directional placement of the spherical satellite AuNPs@A_18_-DNA at the NR tips of the AuNRs@T_18_-DNA@Ag, along with low interparticle distance between both, maximizes the plasmonic coupling, resulting in a prominent and inhomogeneous plasmon broadening at wavelengths >580 nm (see Figure 2B). Figure 2C and Figure 3A show representative TEM micrographs of directional nanoassemblies obtained by this approach, illustrating their fully anisotropic character, i.e., with no AuNPs@A_18_-DNA satellite self-assembled at the lateral facets of the AuNRs@T_18_-DNA@Ag. The overall directional nanoassembly yield was ~52% (as determined from TEM analysis with X_n_, where n is the number of AuNP satellites, as shown in Figure 3B), with ~36% of the directional nanoassemblies featuring 1 AuNP@A_18_-DNA at their tip region, i.e., 1 satellite (X_1_); ~6% of 1 AuNP@A_18_-DNA at each tip, i.e., 2 satellites (X_2_); and ~10% featuring ≥2 AuNPs@A_18_-DNA at their tip region, i.e., 3 satellites (X_3_) (see Figure 3B). The samples contain about 48% of nonassembled building blocks (Figure 3B; X_0_, i.e., 0 satellites) which cannot be easily separated from the assembly due to their similar size and mass (mostly contributed by the building block itself). We note here that due to strong Ag oxidation effects during sample preparation for TEM analysis, it was not possible to obtain TEM micrographs featuring a high concentration of nanoassemblies.

**Figure 4:**
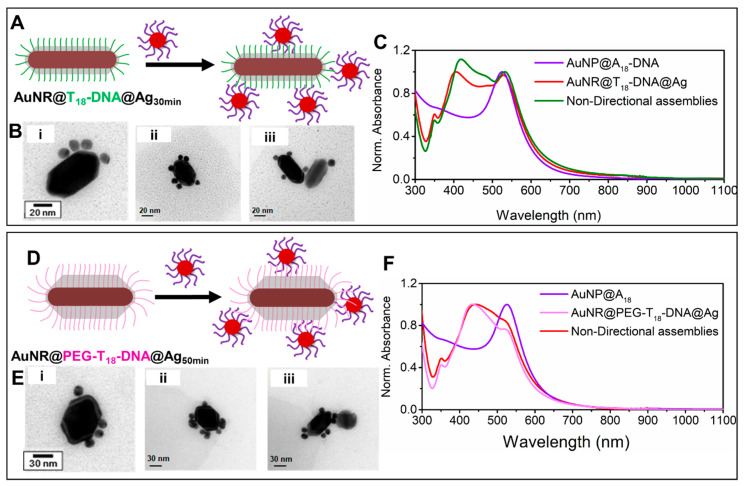
Nondirectional nanoassemblies obtained upon incubation of AuNRs@T_18_-DNA@Ag (overgrown for 30 min, left panel) or of AuNRs@PEG-T_18_-DNA@Ag (overgrown for 50 min, right panel) with AuNPs@A_18_-DNA. (**A**,**D**) Schematic illustration of the formation of nondirectional nanoassemblies for both cases. (**B**,**E**) Representative TEM micrographs of the corresponding nondirectional nanoassemblies. (**C**,**F**) UV–Vis–NIR spectra of the respective building blocks and their resulting nondirectional nanoassemblies.

***Formation of nondirectional nanoassemblies with nondirectional isotropic AuNRs@DNA@Ag as building blocks.*** In contrast, the AuNRs@T_18_-DNA@Ag overgrown for 30 min yielded nondirectional nanoassemblies upon incubation with AuNPs@A_18_-DNA (see Figure 4A–C). In this case, the Ag coating was not thick enough to fully embed and inactivate the T_18_-DNA strands at the lateral facets. The AuNPs@A_18_-DNA could, therefore, hybridize with the available T_18_-DNA strands on the surface of the truncated AuNRs@T_18_-DNA@Ag octahedra. This led to anchoring of the spherical satellite NPs at random positions along their surface, resulting in a nondirectional assembly, with a practically insignificant plasmon broadening and red-shift (Figure 4C, green curve), in spite of their high satellite density. This insignificant plasmon broadening and red-shift is in stark contrast with the remarkable plasmon broadening and red-shift exhibited by the anisotropic directional nanoassemblies (Figure 2B, red spectrum). This suggests that higher hot-spot density, although important for maximizing plasmon coupling and SERS performance, is not the only governing factor. As illustrated by our anisotropic directional plasmonic nano-assemblies, hot-spot positioning plays a far more important role in this case; especially when the hot-spot positioning can be precisely designed to occur at the regions of maximum electric field enhancement, as demonstrated herein. 

As the Ag surface and coating thickness also contribute to the scattering cross-section and plasmon response of the nanoassemblies, we prepared another sample wherein the Ag coating thickness was similar to that of the anisotropic building blocks (Ag coating reaction occurred for about 50 min), with the exception that some of the DNA length was still active at the lateral facets. For this, we functionalized the parent AuNRs@CTAB with a longer ssDNA sequence bearing an additional 12-unit polyethylene glycol (PEG_12_) spacer at the 5′ end (namely HS-C6-PEG_12_-5′-TTTTTTTTTTTTTTTTTT-3′-6-FAM, PEG-T_18_-DNA, estimated length ~19 nm). Silver overgrowth was performed on the AuNRs@PEG-T_18_-DNA, as reported earlier for the AuNRs@T_18_-DNA (Ag^+^/Au = 6) until the reaction was completed (50 min), which yielded AuNRs@PEG-T_18_-DNA@Ag with an average Ag thickness of ~18 ± 3 nm at the lateral facets. Differing from the results shown in Figure 2 for the shorter T_18_-DNA strands, the PEG-T_18_-DNA strands were longer than the average Ag layer thickness at the lateral facets of the AuNRs@PEG-T_18_-DNA@Ag; therefore, most strands were available for hybridization with the AuNPs@A_18_-DNA. The formation of nondirectional nanoassemblies is demonstrated in Figure 4D–F by the corresponding TEM micrographs which exhibits a lack of plasmonic coupling. These results confirm that the Ag shell interacting with the satellite gold NPs is not solely responsible for the significant plasmon coupling at λ > 550 nm observed for the anisotropic nondirectional nanoassemblies. More importantly, it further reconfirms that targeted hot-spot positioning at high electric field enhancement regions (such as the NRs tip region in our directional nanoassemblies) is far more critical than hot-spot density (case of our non-directional nanoassemblies). 

Furthermore, these results demonstrate the versatility of our strategy for preparing directional or nondirectional nanoassemblies, i.e., featuring structural anisotropy or isotropy, by simply adjusting the extent of Ag overgrowth. Apart from the tailored site-selectivity offered by this approach, the obtained sub-100 nm nanoassemblies are very robust against disassembly as a result of the ssDNA strands being effectively buried (locked) within the Ag coating to some extent, even at the tips. This prevents their detachment from the AuNRs’ surface, and therefore drastically reduces the likelihood of disassembly. Furthermore, as this approach only relies on the deployment of simple ssDNA oligonucleotides as assembly linkers, compared to other complex and costly tailor-made DNA nanostructures [22,36], the preparation of such nanoassemblies is rather straightforward and cost-efficient, allowing the process to potentially be scaled up.

**Figure 5:**
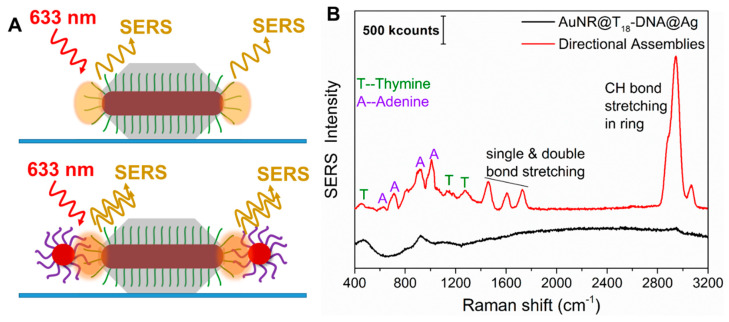
(**A**) Sketch of the anisotropic directional building blocks (AuNRs@T_18_-DNA@Ag_50min_, top) and their anisotropic directional nanoassemblies (bottom). The orange shading highlights the spots of highest electric-field enhancement, i.e., the strategically positioned hot-spots that can lead to SERS of the ssDNA (top panel) or of the dsDNA (bottom panel). (**B**) Representative SERS spectra of a single AuNRs@T_18_-DNA@Ag (directional building block, black curve) and of a single anisotropic directional nanoassembly (red curve), as investigated by dark-field microscope (DFM)-SERS (λ_laser_ = 633 nm). The characteristic SERS peaks from A and T are indicated.

***The roles of ainsotropy, hot-spot positioning, and SERS molecule positioning on SERS performance.*** The observation of plasmon coupling for only the anisotropic directional nanoassemblies established the importance of retaining the anisotropy of the gold nanorod structures in the assemblies. Given the strong plasmonic coupling of our directional nanoassemblies (Figure 2B), we investigated whether their precisely positioned hot-spots could indeed enhance the Raman signals of the interconnecting dsDNA. The inherently low Raman cross-section of the interconnecting adenine (A) and thymine (T) nucleobases, as well as their low concentration at the hot-spots, makes it rather challenging to sufficiently enhance them for detection. However, when such molecules are placed in precisely positioned and intensified hot-spots, detection can be possible. To judge the enhancement of the anisotropic nanoassemblies, we compared the signals of the thymine present in identical concentrations in the anisotropic building blocks with that of their anisotropic directional nanoassemblies. To this end, we spin-coated diluted dispersions of anisotropic building block AuNRs@T_18_-DNA@Ag overgrown for 50 min and their corresponding anisotropic directional nanoassemblies (see Figure 2 and Figure 3A) onto a glass substrate. The SERS performance of both samples was probed in a dark-field microscope (DFM) coupled with a Raman spectrometer using a 633 nm laser for excitation. The very low densities of building blocks and nanoassemblies per μm^2^ on the glass substrates (∼0.15 per μm^2^, determined by SEM) ensured that the detected SERS signals originated from either single building blocks or single nanoassemblies (see sketch in Figure 5A). The SERS spectrum from the AuNRs@T_18_-DNA@Ag (control, Figure 5B—black curve) shows no significant SERS signal from the T nucleobases. It was calculated that ~64 T_18_-DNA strands per tip were present (see detailed calculations in Section S2.1 in Supplementary Materials). In contrast, the anisotropic directional nanoassemblies display well-defined SERS signals from both the A and T nucleobases of the dsDNA (see Figure 5B—red curve; also see additional spectra in Figure S8). The characteristic vibrational modes of adenine at 623 cm^−1^ (NR bending), 724 cm^−1^ (ring stretching), 925 cm^−1^ (NH_2_ ring stretching), and 1007 cm^−1^ (NC stretching) can be clearly observed [37]. The same occurs for the signature peaks of thymine at 456 cm^−1^ (CN bending), 812 cm^−1^ (NC stretching), 1188 cm^−1^ (ring CC stretching and CH bending), and 1275 cm^−1^ (ring stretching) [37]. The intense SERS peaks at ~1500–1700 cm^−1^ and ~2900 cm^−1^ due to CH bond stretching, double bond stretching, and H bending modes are also significantly enhanced by the anisotropic nanoassemblies. The SERS performance, thus, demonstrates that the tailor-made anisotropic directional nanoassemblies featuring strong plasmon coupling combined with the intense electric field at the strategically designed hot-spots at the tips (retaining the anisotropic behavior) and the precise positioning of the SERS molecules (here, A and T) at those intense hot-spots make the interconnecting dsDNA SERS-detectable. Furthermore, if we had assumed that all dsDNA in the NRs’ tip areas were exactly positioned in the hot-spots, such directional nanoassemblies would allow for the detection of ~10 dsDNA, i.e., 180 nucleobases (see calculation details in Section S2.2 in Supplementary Materials). We note here that a direct comparison of SERS results from different works is not trivial due to differences in the nanoparticles or nanostructures employed, as well as the linker type, laser conditions, and tag used. Nevertheless, when comparing SERS signals from short-chain DNA positioned at nanojunctions, our study demonstrates a significantly improved limit of detection of ~10 dsDNA, as compared to ~10^8^ DNA molecules (≡0.893 fM) reported in a study by Guo and co-workers [38].

**Figure 6:**
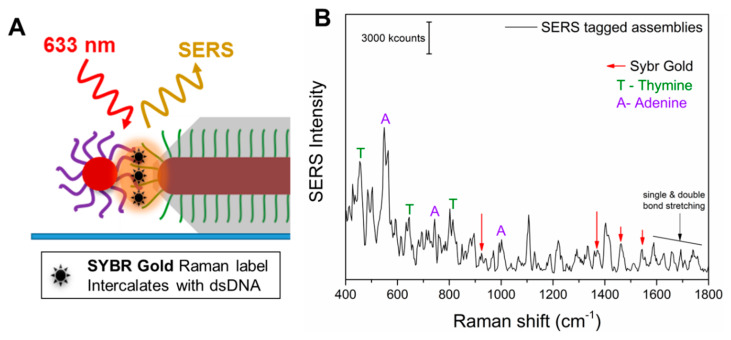
(**A**) Magnified depiction of a SYBR-gold-tagged anisotropic directional nanoassembly spin-coated onto a glass substrate. The SYBR gold intercalates within the dsDNA. The plasmonic hot-spot is depicted in orange. (**B**) Representative SERS spectrum from a SYBR-gold-tagged directional nanoassembly. The signature peaks of adenine (A), thymine (T), and SYBR gold are indicated.

As the A and T of the dsDNA were not exclusively positioned in the hot-spots but were also embedded in the Ag coating and on the surface of the spherical NPs, the importance of the SERS molecule positioning might not be conclusive. Therefore, we carried out further experiments involving the addition of a specific external Raman tag, namely SYBR gold. SYBR gold is a cyanine-based dye that has high affinity for dsDNA and would intercalate between the dsDNA, which is only present at the positioned hot-spots of the anisotropic directional nanoassemblies. Figure 6A shows a sketch of the hot-spot formed due to the assembly (hybridization) of a directional AuNR@T_18_-DNA@Ag and an AuNP@A_18_-DNA, and the SYBR gold molecules intercalated within the interconnecting dsDNA. The SERS spectrum in Figure 6B demonstrates that the SYBR gold tag could be readily detected in the anisotropic nanoassemblies, along with the presence of both A and T, further confirming that the SYBR gold intercalates with the dsDNA and that they all occupy the intense hot-spot. The Raman spectrum of the tag by itself at ~19.6 mM concentration (8 × 10^8^ molecules on the substrate and in the laser spot area, as shown in Section S2.4 in the Supplementary Materials) exhibited a low signal intensity, even at such a high concentration (see Figure S9 and Section S2.3 in Supplementary Materials). In contrast, the individual anisotropic directional nanoassemblies allow for comparable SERS intensities from approximately 47 SYBR gold molecules (see Figure 6B, Figure S10, and Section S2.4 in Supplementary Materials for the calculations) due to their significant SERS enhancement, as a result of being well positioned in the intense hot-spots of the anisotropic directional nanoassemblies. This allowed us to determine the average enhancement factor of the individual anisotropic nanoassemblies, which was ~1.6 × 10^8^ (see Section S2.4 Supplementary Materials). Such a significant SERS enhancement is due to the effective intercalation of the SYBR gold Raman tags in the precisely positioned intense hot-spots of the anisotropic directional nanoassemblies. This remarkable enhancement factor also explains the excellent SERS performance of such anisotropic nanoassemblies in the tag-free SERS experiments presented earlier. In contrast, the nondirectional nanoassemblies, when investigated for their SERS signal, provided a significantly lower average signal intensity than that observed for the tagged directional nanoassemblies. The low average signal intensity was accompanied by a negligible signal (SNR < 2) in some cases, thereby resulting in a high standard deviation in SERS enhancement factor (EF) for both types of nondirectional nanoassemblies. This high standard deviation is probably caused by the random and varied positioning of the satellites and hot-spots, which can be observed in Figure 4B,E. Such observations emphasize the crucial importance of carefully designing the hot-spot positions, placing the SERS molecules in those hot-spots, in addition to the hot-spot density and colloidal stability of the nanoassemblies. It also suggests that although we appreciate the high plasmon response of the shaped nanostructures, such as nanorods, we would need to maintain those properties (such as the anisotropic positioning of the hot-spots of the nanorods) in the nanoassemblies in order to benefit from the unique optical properties of the shaped nanostructures. This would thusballow researchers to extract maximized plasmon and SERS responses. Overall, given the extraordinary SERS performance of the directional anisotropic nanoassemblies presented herein, they could be envisioned as ultrasensitive and photostable SERS agents for nano-, bio-, and chemosensing.

## 4. Conclusions

In summary, we have shown that in order to maximize plasmon coupling and SERS responses from nanoassemblies, it is very important to carefully control the position of the hot-spots responsible for the highest electric field enhancements. We successfully report the synthesis of directional anisotropic, nanosized assemblies featuring strong plasmon coupling obtained by designing AuNRs@DNA building blocks with site-selective DNA interactions at their tips. Such anisotropic directional building blocks were obtained using a controlled DNA inactivation strategy by embedding the ssDNA upon controlled Ag overgrowth on the surface of single-crystal AuNRs@DNA. We have shown that for gold nanorod core and NP satellite assembly morphologies, anisotropic directional assemblies with NP–NP junction hot-spots precisely psoitioned only at the tips show dramatically increased plasmon coupling in the Vis–NIR range as compared to nondirectional assemblies. This leads to a strong SERS enhancement at the tip-positioned hot-spots, both in: (a) the tag-free scenario, where the interconnecting dsDNA nucleobases typically of low Raman cross-sections are detectable, with a detection limit of ~10 interconnecting dsDNA strands equivalent to 180 nucleobases; and (b) the SYBR-gold-tagged scenario, intercalating only with the dsDNA present in the intense hot-spots, featuring a SERS enhancement of ~1.6 × 10^8^. This understanding of the important roles of hot-spot positioning and SERS molecule positioning, along with the reported strategy, might provide solutions to further enhance plasmonic and SERS responses, which are crucial due to the ever-growing applications of nanoplasmonics in solving real-life detection problems.

## Supplementary Materials

The following are available online at https://www.mdpi.com/2079-4991/10/5/942/s1: Figures S1–S7: Demonstrating the effect of different conditions on the Ag overgrowth process of AuNR@DNA with UV-Vis spectra and TEM images, Figure S8: SERS spectra of two individual directional nano-assemblies, Figure S9: Raman spectrum of SYBR Gold and Figure S10: Representative SERS spectra from two SYBR gold-tagged directional nano-assemblies. Additional experimental optimization studies on the formation of directional AuNRs@DNA@Ag, and further SERS results and calculations.

## References

[1] Vo-dinh T., Liu Y., Crawford B.M., Wang H., Yuan H., Register J.K., Zhang Y., Liu Z.Z., Thackray B.D., Bao Z. (2017). Rapid, One-Pot, Protein-Mediated Green Synthesis of Gold Nanostars for Computed Tomographic Imaging and Photothermal Therapy of Cancer. Chem. Soc. Rev..

[2] Guerrini L., Graham D. (2012). Molecularly-Mediated Assemblies of Plasmonic Nanoparticles for Surface-Enhanced Raman Spectroscopy Applications. Chem. Soc. Rev..

[3] Sajanlal P.R., Sreeprasad T.S., Samal A.K., Pradeep T. (2011). Anisotropic Nanomaterials: Structure, Growth, Assembly, and Functions. Nano Rev..

[4] Pansare V., Hejazi S., Faenza W., Prud’homme R.K. (2012). Review of Long-Wavelength Optical and NIR Imaging Materials: Contrast Agents, Fluorophores and Multifunctional Nano Carriers. Chem. Mater..

[5] Dey P., Tabish T.A., Mosca S., Palombo F., Matousek P., Stone N. (2020). Plasmonic Nanoassemblies: Tentacles Beat Satellites for Boosting Broadband NIR Plasmon Coupling Providing a Novel Candidate for SERS and Photothermal Therapy. Small.

[6] Jin R. (2010). Nanoparticle Clusters Light Up in SERS. Angew. Chem. Int. Ed..

[7] Laing S., Jamieson L.E., Faulds K., Graham D. (2017). Surface-Enhanced Raman Spectroscopy for In Vivo Biosensing. Nat. Rev. Chem..

[8] Taylor R.W., Esteban R., Mahajan S., Aizpurua J., Baumberg J.J. (2016). Optimizing SERS from Gold Nanoparticle Clusters: Addressing the Near Field by an Embedded Chain Plasmon Model. J. Phys. Chem. C.

[9] Zhou W., Gao X., Liu D., Chen X. (2015). Gold Nanoparticles for in Vitro Diagnostics. Chem. Rev..

[10] Couture M., Liang Y., Poirier Richard H.-P., Faid R., Peng W., Masson J.-F. (2013). Tuning the 3D Plasmon Field of Nanohole Arrays. Nanoscale.

[11] Alvarez-Puebla R.A., Liz-Marzan L.M. (2010). SERS-Based Diagnosis and Biodetection. Small.

[12] Guerrini L., Alvarez-Puebla R.A. (2019). Surface-Enhanced Raman Spectroscopy in Cancer Diagnosis, Prognosis and Monitoring. Cancers (Basel).

[13] Golovynskyi S., Golovynska I., Stepanova L.I., Datsenko O.I., Liu L., Qu J., Ohulchanskyy T.Y. (2018). Optical Windows for Head Tissues in Near-Infrared and Short-Wave Infrared Regions: Approaching Transcranial Light Applications. J. Biophotonics.

[14] Tabish T.A., Dey P., Mosca S., Salimi M., Palombo F., Matousek P., Stone N. (2020). Smart Gold Nanostructures for Light Mediated Cancer Theranostics: Combining Optical Diagnostics with Photothermal Therapy. Adv. Sci..

[15] Klinkova A., Choueiri R.M., Kumacheva E. (2014). Self-Assembled Plasmonic Nanostructures. Chem. Soc. Rev..

[16] Jones M.R., Osberg K.D., Macfarlane R.J., Langille M.R., Mirkin C.A. (2011). Templated Techniques for the Synthesis and Assembly of Plasmonic Nanostructures. Chem. Rev..

[17] Dey P., Zhu S., Thurecht K.J., Fredericks P.M., Blakey I. (2014). Self Assembly of Plasmonic Core-Satellite Nano-Assemblies Mediated by Hyperbranched Polymer Linkers. J. Mater. Chem. B.

[18] Dey P., Blakey I., Thurecht K.J., Fredericks P.M. (2013). Self-Assembled Hyperbranched Polymer-Gold Nanoparticle Hybrids: Understanding the Effect of Polymer Coverage on Assembly Size and SERS Performance. Langmuir.

[19] Hüsken N., Taylor R.W., Zigah D., Taveau J.-C., Lambert O., Scherman O.A., Baumberg J.J., Kuhn A. (2013). Electrokinetic Assembly of One-Dimensional Nanoparticle Chains with Cucurbit[7]Uril Controlled Subnanometer Junctions. Nano Lett..

[20] Rothemund P.W.K. (2006). Folding DNA to Create Nanoscale Shapes and Patterns. Nature.

[21] Kuhler P., Roller E.M., Schreiber R., Liedl T., Lohmuller T., Feldmann J. (2014). Plasmonic DNA-Origami Nanoantennas for Surface-Enhanced Raman Spectroscopy. Nano Lett..

[22] Thacker V.V., Herrmann L.O., Sigle D.O., Zhang T., Liedl T., Baumberg J.J., Keyser U.F. (2014). DNA Origami Based Assembly of Gold Nanoparticle Dimers for Surface-Enhanced Raman Scattering. Nat. Commun..

[23] Tan L.H., Xing H., Chen H., Lu Y. (2013). Facile and Efficient Preparation of Anisotropic DNA-Functionalized Gold Nanoparticles and Their Regioselective Assembly. J. Am. Chem. Soc..

[24] Xu L., Kuang H., Xu C., Ma W., Wang L., Kotov N.A. (2012). Regiospecific Plasmonic Assemblies for in Situ Raman Spectroscopy in Live Cells. J. Am. Chem. Soc..

[25] Dey P., Thurecht K.J., Fredericks P.M., Blakey I. (2019). Tagged Core-Satellite Nanoassemblies: Role of Assembling Sequence on Surface-Enhanced Raman Spectroscopy (SERS) Performance. Appl. Spectrosocpy.

[26] Liu M., Guyot-Sionnest P. (2005). Mechanism of Silver(I)-Assisted Growth of Gold Nanorods and Bipyramids. J. Phys. Chem. B.

[27] Nikoobakht B., El-Sayed M.A. (2003). Preparation and Growth Mechanism of Gold Nanorods (NRs) Using Seed-Mediated Growth Method. Chem. Mater..

[28] Baumann V., Röttgermann P.J.F., Haase F., Szendrei K., Dey P., Lyons K., Wyrwich R., Gräßel M., Stehr J., Ullerich L. (2016). Highly Stable and Biocompatible Gold Nanorod–DNA Conjugates as NIR Probes for Ultrafast Sequence-Selective DNA Melting. RSC Adv..

[29] Zipper H., Brunner H., Bernhagen J., Vitzthum F. (2004). Investigations on DNA Intercalation and Surface Binding by SYBR Green I, Its Structure Determination and Methodological Implications. Nucleic Acids Res..

[30] Sanchez-Iglesias A., Carbo-Argibay E., Glaria A., Rodriguez-Gonzalez B., Perez-Juste J., Pastoriza-Santos I., Liz-Marzan L.M. (2010). Rapid Epitaxial Growth of Ag on Au Nanoparticles: From Au Nanorods to Core-Shell Au@Ag Octahedrons. Chem. Eur. J..

[31] Ma Y., Li W., Cho E.C., Li Z., Yu T., Zeng J., Xie Z., Xia Y. (2010). Au@Ag Core-Shell Nanocubes with Finely Tuned and Well- Controlled Sizes, Shell Thicknesses, and Optical Properties. ACS Nano.

[32] Chen H., Yuan Z., Wu C. (2015). Nanoparticle Probes for Structural and Functional Photoacoustic Molecular Tomography. Biomed Res. Int..

[33] Lee J.H., Kim G.H., Nam J.M. (2012). Directional Synthesis and Assembly of Bimetallic Nanosnowmen with DNA. J. Am. Chem. Soc..

[34] Jiang R., Chen H., Shao L., Li Q., Wang J. (2012). Unraveling the Evolution and Nature of the Plasmons in (Au Core)-(Ag Shell) Nanorods. Adv. Mater..

[35] Olson W.K. (1975). Configurational Statistics of Polynucleotide Chains. A Single Virtual Bond Treatment. Macromolecules.

[36] Shen C., Lan X., Lu X., Meyer T.A., Ni W., Ke Y., Wang Q. (2016). Site-Specific Surface Functionalization of Gold Nanorods Using DNA Origami Clamps. J. Am. Chem. Soc..

[37] Otto C., van den Tweel T.J.J., de Mu F.F.M., Greve J. (1986). Surface-Enhanced Raman Spectroscopy of DNA Bases. J. Raman Spectrosc..

[38] Guo R., Yin F., Sun Y., Mi L., Shi L., Tian Z., Li T. (2018). Ultrasensitive Simultaneous Detection of Multiplex Disease-Related Nucleic Acids Using Double-Enhanced Surface-Enhanced Raman Scattering Nanosensors. ACS Appl. Mater. Interfaces.

